# Electronic structures and unusually robust bandgap in an ultrahigh-mobility layered oxide semiconductor, Bi_2_O_2_Se

**DOI:** 10.1126/sciadv.aat8355

**Published:** 2018-09-14

**Authors:** Cheng Chen, Meixiao Wang, Jinxiong Wu, Huixia Fu, Haifeng Yang, Zhen Tian, Teng Tu, Han Peng, Yan Sun, Xiang Xu, Juan Jiang, Niels B. M. Schröter, Yiwei Li, Ding Pei, Shuai Liu, Sandy A. Ekahana, Hongtao Yuan, Jiamin Xue, Gang Li, Jinfeng Jia, Zhongkai Liu, Binghai Yan, Hailin Peng, Yulin Chen

**Affiliations:** 1 Department of Physics, University of Oxford, Oxford OX1 3PU, UK.; 2 School of Physical Science and Technology, ShanghaiTech University and Chinese Academy of Sciences–Shanghai Science Research Center, 393 Middle Huaxia Road, Shanghai 201210, People’s Republic of China.; 3 Center for Nanochemistry, Beijing National Laboratory for Molecular Sciences, College of Chemistry and Molecular Engineering, Peking University, Beijing 100871, People’s Republic of China.; 4 Department of Condensed Matter Physics, Weizmann Institute of Science, Rehovot 7610001, Israel.; 5 Max Planck Institute for Chemical Physics of Solids, D-01187 Dresden, Germany.; 6 State Key Laboratory of Low Dimensional Quantum Physics, Department of Physics, Tsinghua University, Beijing 100084, People’s Republic of China.; 7 Advanced Light Source, Lawrence Berkeley National Laboratory, Berkeley, CA 94720, USA.; 8 Accelerator Laboratory, Pohang University of Science and Technology, Pohang 790-784, Korea.; 9 Paul Scherrer Institute, 5232 Villigen, Switzerland.; 10 National Laboratory of Solid-State Microstructures, College of Engineering and Applied Sciences, and Collaborative Innovation Center of Advanced Microstructures, Nanjing University, Nanjing 210093, People’s Republic of China.; 11 Key Laboratory of Artificial Structures and Quantum Control (Ministry of Education), Department of Physics and Astronomy, Shanghai Jiao Tong University, Shanghai 200240, People’s Republic of China.

## Abstract

Semiconductors are essential materials that affect our everyday life in the modern world. Two-dimensional semiconductors with high mobility and moderate bandgap are particularly attractive today because of their potential application in fast, low-power, and ultrasmall/thin electronic devices. We investigate the electronic structures of a new layered air-stable oxide semiconductor, Bi_2_O_2_Se, with ultrahigh mobility (~2.8 × 10^5^ cm^2^/V⋅s at 2.0 K) and moderate bandgap (~0.8 eV). Combining angle-resolved photoemission spectroscopy and scanning tunneling microscopy, we mapped out the complete band structures of Bi_2_O_2_Se with key parameters (for example, effective mass, Fermi velocity, and bandgap). The unusual spatial uniformity of the bandgap without undesired in-gap states on the sample surface with up to ~50% defects makes Bi_2_O_2_Se an ideal semiconductor for future electronic applications. In addition, the structural compatibility between Bi_2_O_2_Se and interesting perovskite oxides (for example, cuprate high–transition temperature superconductors and commonly used substrate material SrTiO_3_) further makes heterostructures between Bi_2_O_2_Se and these oxides possible platforms for realizing novel physical phenomena, such as topological superconductivity, Josephson junction field-effect transistor, new superconducting optoelectronics, and novel lasers.

## Teaser

A new layered oxide semiconductor (Bi_2_O_2_Se) is found with excellent electronic properties for promising applications.

## INTRODUCTION

The search for new materials with superior electronic properties is critical to the development and prosperity of the semiconductor industry. In the past decade, two-dimensional (2D) materials [for example, graphene (*1*–*3*), transition metal dichalcogenides (TMDs) (*4*–*8*), and black phosphorus (*4*, *9*, *10*)] have grown as promising candidates with great potential for future electronic applications, especially those with high carrier mobility, moderate bandgap, and ambient environment stability, and numerous 2D materials have been intensively investigated. Graphene, for example, is a robust atomically thin 2D carbon sheet with ultrahigh carrier mobility (>10,000 cm^2^/V⋅s at room temperature) (*1*, *3*). However, the lack of a sizeable bandgap (zero gap in monolayer graphene and very small gap for multilayer graphene) (*11*, *12*) limits its application in field-effect devices. Few-layer TMDs, on the other hand, exhibit sizable bandgap (for example, 1.8 eV for monolayer MoS_2_), but their application is restricted by the relatively low carrier mobility (typically less than 100 cm^2^/V·s for MoS_2_ thin flakes at room temperature) (*5*–*7*). Recently, few-layer black phosphorus has emerged as a good 2D semiconductor candidate, with both appreciable thickness-dependent bandgap (0.3 to 2.0 eV from bulk to monolayer) and relatively high carrier mobility [~1000 cm^2^/V⋅s at room temperature (*9*)], but its metastability at ambient environment (*13*) has hindered its potential for broad application. Therefore, the search for 2D semiconductors with excellent electronic performance and stability in the ambient environment remains urgent.

More recently, Bi_2_O_2_Se, an air-stable layered oxide, has emerged as a promising new semiconductor with excellent electronic properties. Its layered nature makes it ideal for fabricating electronic devices down to a few atomic layers (even monolayer), which is demonstrated in a recent study (*14*). The Bi_2_O_2_Se-based top-gated field-effect transistor device shows excellent semiconductor device properties, including high carrier mobility (~28,900 cm^2^/V⋅s at 1.9 K and ~450 cm^2^/V⋅s at room temperature) and superior current on/off ratio of >10^6^ with almost ideal subthreshold swing (~65 mV/dec). In addition, the moderate bandgap (~0.8 eV) of Bi_2_O_2_Se makes its device suitable for room temperature operation while requiring only a relatively low operation voltage [for example, compare to Si with a 1.17-eV bandgap (*15*)]. These attractive properties, together with its stability in the ambient environment and easy accessibility (bulk crystal, thin film, and nanostructures are all readily accessible), make Bi_2_O_2_Se a promising semiconductor candidate for future ultrasmall high-performance and low-power electronic devices.

Besides its potential in electronic applications, Bi_2_O_2_Se is also a thermoelectric material (*16*), with the thermoelectric figure-of-merit *ZT* predicted as high as 1.42 (comparable to Bi_2_Te_3_, one of the best thermoelectric materials broadly used today) if an in-plane strain is applied (*17*). Moreover, as the Bi-O layer in Bi_2_O_2_Se is structurally compatible with many perovskite oxides that exhibit rich interesting physical phenomena (for example, ferroelectricity, magnetism, multiferroics, and high-*T*_c_ superconductivity), it is possible to fabricate hybrid structures/superlattices between Bi_2_O_2_Se and various perovskite oxides [for example, Bi_2_Sr_2_Ca_*n*−1_Cu_*n*_O_2*n*+4+*x*_ series high–transition temperature superconductors (HTSCs) and SrTiO_3_] to pursue novel emergent physical phenomena in hybrid semiconductor-superconductor heterostructures, such as topological superconductivity, Josephson junction field-effect transistor, new superconducting optoelectronics, and novel lasers (*18*–*22*).

To realize the full potential of Bi_2_O_2_Se and explore its applications in electronic, thermoelectric, and optoelectronic devices, understanding its detailed electronic structures is essential. For this purpose, we combined angle-resolved photoemission spectroscopy (ARPES) and scanning tunneling microscopy (STM) to systematically map out the full band structure of Bi_2_O_2_Se with key parameters including the effective mass, Fermi velocity, and the bandgap. The bandgap from both ARPES and STM shows unusual robustness and spatial uniformity without undesired in-gap (surface or edge) states—even on the cleavage sample surface with up to ~50% of Se deficiency—making Bi_2_O_2_Se an ideal semiconductor for future electronic applications.

## RESULTS

### Basic characterizations

**Fig. 1:**
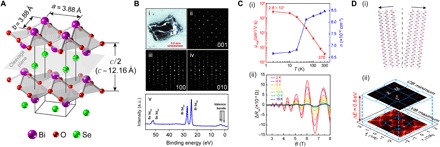
General characterizations of Bi_2_O_2_Se single crystals. (**A**) Body-center tetragonal crystal structure of Bi_2_O_2_Se, consisting of alternating Bi_2_O_2_ and Se layers. (**B**) (i) Optical image of a Bi_2_O_2_Se single crystal, showing the layered structure and shining cleaved surface. (ii to iv) XRD pattern of the (001), (100), and (010) surfaces, respectively. (v) Core-level photoemission spectrum, showing the characteristic peaks of Bi_5d_ and Se_3d_ levels. a.u., arbitrary units. (**C**) (i) Hall mobility (μ_hall_) and carrier density (*n*) as a function of temperature in Bi_2_O_2_Se single crystal. (ii) SdH oscillatory part of the longitudinal magnetoresistance as a function of applied perpendicular magnetic field (the non-oscillatory background has been removed). (**D**) (i) Illustration of the cleavage process, leaving half Se atoms attached to each Bi_2_O_2_ layer (see text for more discussion). (ii) ARPES broad contour maps of conduction band (CB) minimum and valence band (VB) maximum, with the Brillouin zone (BZ) overlapped (blue frames). The indirect bandgap (~0.8 eV) is indicated.

Bi_2_O_2_Se crystallizes into a body-centered tetragonal structure (*I*4/*mmm*, no. 139; *a* = *b* = 3.88 Å, *c* = 12.16 Å) with a repeating sequence of …-(Bi_2_O_2_)_1_-Se_1_-(Bi_2_O_2_)_2_-Se_2_-… layers, as illustrated in Fig. 1A. High-quality Bi_2_O_2_Se single bulk crystals (Fig. 1B, i) for this study were synthesized by a modified Bridgman method (see Materials and Methods for details), as verified by the x-ray diffraction (XRD) characterization (Fig. 1B, ii to iv) and the core-level photoemission spectrum (Fig. 1B, v). The Hall measurements of Bi_2_O_2_Se devices show a very high residual-resistance ratio of 585 (*R*_*xx*,300K_/*R*_*xx*,2K_; see fig. S1A) and a superior Hall mobility of 2.8 × 10^5^cm^2^/V⋅s at 2 K (Fig. 1C, i). We note that the metallic behavior in fig. S1A is caused by the residual carriers, which can be removed by electric gating, as demonstrated in (*14*). Besides, prominent Shubnikov–de Haas (SdH) quantum oscillations are also observed at low temperature (Fig. 1C, ii), indicating the long mean free path of the carriers.

In the ARPES and STM investigations, Bi_2_O_2_Se single crystals were cleaved inside the ultrahigh vacuum (UHV) chambers for in situ measurements. Because of the weak interaction between Bi_2_O_2_ and Se layers (see Fig. 1, A and D, i), the cleavage occurs on the Se plane, leaving 50% Se atoms attached to each Bi_2_O_2_ plane, as required by the charge neutral requirement and considering that the two Bi_2_O_2_ layers are symmetric on each side of the Se plane. The resulting cleaved sample surface shows an interesting intertwined weave pattern formed by Se atoms and vacancies in our STM study, which will be discussed in detail later. Surprisingly, for such a high percentage of surface defects (~50% vacancies), the ARPES measurements (Fig. 1D, ii) show a clean bandgap (indirect, ~0.8 ± 0.05 eV) between the conduction and valance bands without signatures of undesired in-gap states detrimental for the device applications (*23*, *24*).

### Scanning tunneling spectroscopy measurements

**Fig. 2:**
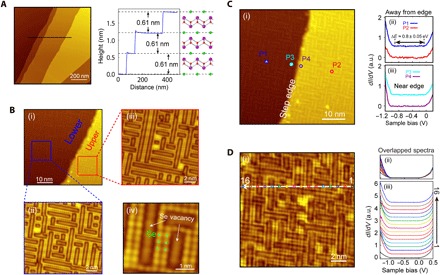
Surface morphology and uniform bandgap. (**A**) Large-scale STM scan on the cleaved Bi_2_O_2_Se surface (left) showing clear terraces with step edges of ~0.61 nm in height (right). (**B**) (i) Zoomed-in STM image in the vicinity of a step edge. (ii and iii) Atomic-resolution surface topography on lower and upper terraces, respectively. Both shows intertwined weave patterns formed by ~50% Se vacancies. (iv) Further zoomed-in image illustrates the Se atoms and Se vacancies. (**C**) STS spectra in the vicinity and away from a step edge, showing a ~0.85 ± 0.05 eV of bandgap free from the undesired surface or edge in-gap states. (**D**) STS spectra taken from 16 consecutive surface locations [along a line indicated in (i)] show the uniformity of the gap size without in-gap states in (ii) and (iii), respectively. (ii) Overlapped STS spectra to demonstrate the uniform gap size. (iii) Offset STS spectra for clarity.

To confirm the nonexistence of undesired in-gap (surface or edge) states, we performed extensive STM investigations on the cleaved sample surfaces, as summarized in Fig. 2. The layered nature of Bi_2_O_2_Se is evident from the topography map (Fig. 2A), which shows large flat terraces with a step height of ~0.61 nm (*c*/2). The zoom-in STM measurements on upper and lower terraces (Fig. 1B) both illustrate the intertwined weave pattern formed by the Se vacancies that constitute ~50% of the total surface area (more details and the statistical results from larger-area STM measurements can be found in the Supplementary Materials). The Se atoms and vacancies in Fig. 2B show obvious dimerization for both Se atoms and vacancies, as we will discuss in detail later.

To investigate the uniformity of the bandgap of Bi_2_O_2_Se, we then carried out scanning tunneling spectroscopy (STS) studies (Fig. 2, C and D). Regardless where the STS spectra were taken—from either the points far away from the step edge (for example, points P1 and P2 in Fig. 2C, i) or the points at the vicinity of the step edge (for example, points P3 and P4 in Fig. 2C, i)—the *dI*/*dV* spectra all show clean bulk bandgap (~0.85 ± 0.05 eV) without any observable in-gap states, as illustrated in Fig. 2C (ii and iii). The missing in-gap states are more evident in Fig. 2D, where the *dI*/*dV* spectra were taken from 16 consecutive surface positions (Fig. 2D, i), on top of either Se atoms or vacancies.

### ARPES measurements

Besides the STM studies, systematic ARPES experiments were performed to reconstruct the full band structures in the complete 3D BZ. For this purpose, photon energy–dependent ARPES measurements (*25*, *26*) were carried out across a wide range (60 to 230 eV) of photon energy to cover multiple BZs along the *k*_*z*_ direction.

**Fig. 3:**
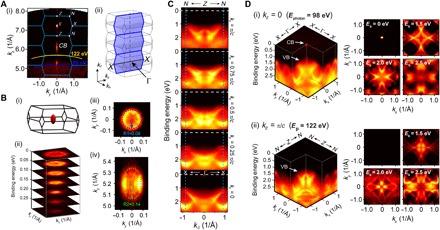
Complete band structure of Bi_2_O_2_Se. (**A**) (i) FS of the *k*_*y*_-*k*_*z*_ plane showing a pocket centered at Г with the long axis along the *z* direction. The blue frames (overlapped) show the *k*_*y*_-*k*_*z*_ BZs, as illustrated in (ii). (**B**) Details of the electron pocket formed by the conduction band. (i) Calculated ellipsoidal FS in the 3D BZ. (ii) Stacking plot of constant energy maps showing the parabolic dispersions determined by the effective mass of the conducting electron pocket. (iii and iv) FS of the electron pocket projected onto the *k*_*x*_-*k*_*y*_ and *k*_*y*_-*k*_*z*_ planes, respectively, showing a nearly isotropic circular shape in (ii) and an ellipse in the *k*_*y*_-*k*_*z*_ plane, consistent with the shape of the calculation in (i). (**C**) Band dispersion plots from different *k*_*z*_ momenta, from *X*-Г-*X* direction (*k*_*z*_ = 0; bottom) to *N*-*Z*-*N* direction (*k*_*z*_ = π/*c*; top). No in-gap states can be seen in all plots. (**D**) Detailed full 3D plots of the band structures for *k*_*z*_ = 0 (*E*_photon_ = 98 eV) and *k*_*z*_ = π/*c* (*E*_photon_ = 122 eV) photons, respectively. Constant energy contours showing the band structures at different binding energies are also illustrated (on the right), indicating difference for the two different *k*_*z*_ values in (i) and (ii).

As can be seen in Fig. 3A, the Fermi surface (FS) map on the *k*_*y*_-*k*_*z*_ plane (together with the FS map of the *k*_*x*_-*k*_*y*_ plane in Fig. 1F) confirms that there is only one electron pocket around the Г point of the BZ, which shows an ellipsoidal shape (Fig. 3B, i) with a nearly isotropic small in-plane (*k*_*x*_-*k*_*y*_) electron mass of *m*_∥_ = (0.14 ± 0.02)*m*_*e*_ and an in-plane Fermi velocity as *V*_F_ = (1.69 ± 0.01) × 10^6^ m/s. Similarly, the parameters of the hole pocket near the VBM can also be deduced from our measurements (see the Supplementary Materials for details), yielding an in-plane hole mass of *m*_*x*_ = (− 2.41 ± 0.02)*m*_*e*_ along the Г-*X* direction and *m*_*y*_ = (− 0.30 ± 0.02)*m*_*e*_ along the *X*-*M* direction. Again, from the band structures in the full 3D BZ, there is no signature of the in-gap states at any *k*_*x*_, *k*_*y*_, or *k*_*z*_ momentum [see Fig. 3, C and D, for examples of dispersions’ evolution at different *k*_*z*_ momenta and the full band structures along the *k*_*x*_-*k*_*y*_ plane for Г (*k*_*z*_ = 0) and *Z* (*k*_*z*_ = π/*c*) points, respectively].

### Surface topography and the robust bandgap

**Fig. 4:**
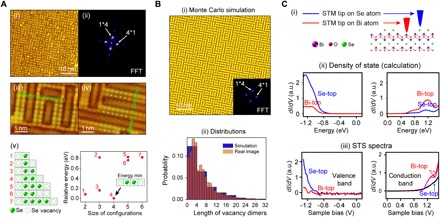
Surface pattern formation and influence on band structure. (**A**) (i) STM image of cleaved Bi_2_O_2_Se surface illustrates a clear intertwined weave pattern. (ii) FFT of (i) shows a period of 4 × Se-Se distance along two orthogonal directions. (iii and iv) Atomically resolved STM images show Se atoms, and vacancies are dimerized along two orthogonal directions, forming periodic patterns with a period of 4 × Se-Se distance. (v) Different Se-atom and vacancy configurations (left) and the calculated formation energies (right). Se-Se dimer with one vacancy on each side has the lowest energy (right) and is thus most favorable, in agreement with the observation in (ii) to (iv). (**B**) (i) Monte Carlo simulation and the FFT pattern of the 50% Se–50% vacancy surface pattern using the most favorable configuration obtained from (A, v) (along both *x* and *y* directions) agree well with the measurements in (A). (ii) Statistics of the characteristic surface feature (distribution of the length of the vacancy dimers) from simulation and measurements exhibit excellent agreement. (**C**) (i) Illustration of STS measurements on Bi and Se atoms on the surface. (ii) Calculated STS (*dI*/*dV*) spectra of Bi and Se terminations showing no in-gap states but with different DOS on the conduction and valence bands. (iii) STS measurements show good consistency with calculation in (ii).

After establishing the overall band structures of Bi_2_O_2_Se and confirming that there are no in-gap states from both ARPES and STM measurements, we now focus on the interesting half Se-covered sample surface and investigate why the massive amount of defects (~50% vacancies) does not give rise to in-gap states. For this purpose, in Fig. 4A (i to iv), we show large-scale atomic-resolution STM topography maps of the sample surface. The brightest spot in the fast Fourier transformation (FFT) of the topography map shows an obvious period of 4 × Se-Se atomic distance (Fig. 4A, ii), and both Se atoms and the vacancies dimerize and form 2 × *n* structures (where *n* is an integer; see Fig. 4A, iii and iv). To understand these results, we carried out ab initio calculations (using a slab model; see the Supplementary Materials for details) to estimate the formation energy of different Se-atom and vacancy configurations. Our analysis (Fig. 4A, v) shows that the chain of Se-Se dimer with one vacancy on each side (that is, …V-Se-Se-V…) with the period of 4 × Se-Se atomic distance is the most energetically favorable, thus naturally explaining the experimental observation in Fig. 4A (i to iv). By using this configuration along both *x* and *y* directions, our Monte Carlo simulation (more details can be found in the Supplementary Materials) gives results markedly similar (Fig. 4B, i) to the STM measurements (Fig. 4A), and the excellent agreement of the statistics of the surface features (for example, the length of vacancy dimers; see. Fig. 4B, ii) again affirms the validity of our model.

By ab initio calculations, we can further investigate the density of state (DOS) from different terminating atoms on the surface (Fig. 4C, i)—from either the Se atom or the Bi atom under the Se vacancy (the calculation details can be found in the Supplementary Materials)—to compare with the STS measurements. Our calculations (Fig. 4C, ii) show that the Se vacancies do not introduce in-gap states; rather, they only affect the DOS in the conduction and valence bands, agreeing well with our STS measurements (Fig. 4C, iii).

The reason why even such a massive amount of surface defects do not introduce in-gap states can be understood in an intuitive picture. In contrast to traditional semiconductors (for example, Si or GaAs) where lattice atoms usually form covalent bonds, Bi_2_O_2_Se is an ionic system consisting of [Bi_2_O_2_]^2+^ and Se^2−^ in adjacent layers. As a result, the Se vacancies behave differently from ordinary donor defects, because removing the Se atoms will not break any covalent bonds. Therefore, these Se defects cause rather high energy levels instead of shallow in-gap states due to the difficulty of negatively charged Se layer in trapping free electrons [more details can be found in a recent theoretical work (*27*)].

## DISCUSSION

The robustness of the bandgap against the surface defects (~50% Se vacancies), together with the small carrier mass, moderate bandgap size, layered nature, and air stability, makes Bi_2_O_2_Se an ideal semiconductor for future electronic device applications, and the tunability of the electronic properties (such as the bandgap size) with thickness (*14*) further makes the application of Bi_2_O_2_Se versatile. The systematic experimental and theoretical studies on the electronic structures of Bi_2_O_2_Se in this work not only gives us a complete understanding of this new semiconductor material but also motivates future studies on the Bi_2_O_2_X (X = S, Se, Te) family of semiconductors.

Furthermore, besides its potential in device applications, Bi_2_O_2_Se also has great potential in fundamental research. For example, the Bi layer in Bi_2_O_2_Se forms a 2D square lattice with a Bi-Bi distance of 3.88 Å, identical to that in the Bi_2_Sr_2_Ca_*n*−1_Cu_*n*_O_2*n*+4+*x*_ series of unconventional HTSC, and Bi_2_O_2_Se is also lattice-matched with the commonly used substrate SrTiO_3_ (cubic phase; lattice constant, 3.9 Å). This lattice matching makes it possible to fabricate Bi_2_O_2_Se-HTSC hybrid junctions and novel interface 2D electron gas (for example, in Bi_2_O_2_Se-SrTiO_3_ heterostructure), thus opening the door for the study of numerous novel phenomena, such as topological superconductivity (*18*), Josephson junction field-effect transistor (*19*), new superconducting optoelectronics (*20*, *21*), and novel lasers (*22*).

## MATERIALS AND METHODS

### Sample synthesis

Bi_2_O_2_Se single crystals were prepared via a modified Bridgman method. The stoichiometric high-purity Bi_2_O_3_ powder (99.999%), Se powder (99.999%), and Bi powder (99.999%) were weighted into evacuated quartz tube with pressure down to 10^−2^ Pa. The Bi_2_O_2_Se powder was obtained when the temperature was kept at 773 K for 6 hours. As-synthesized Bi_2_O_2_Se was grounded into powder, reencapsulated into an evacuated quartz tube, molten at 1223 K for 2 hours, then slowly cooled down to 1123 K for 24 hours, and finally cooled down to room temperature to form a bulk crystal, which is confirmed as tetragonal Bi_2_O_2_Se with the space group of *I*4/*mmm* (*a* = *b* = 3.88 Å, *c* = 12.16 Å, *Z* = 2) by XRD.

### Angle-resolved photoemission spectroscopy

The ARPES experiments were performed on the Bi_2_O_2_Se bulk crystals at beamline I05 of the Diamond Light Source. The data were recorded using a Scienta R4000 analyzer with the total convolved energy and angle resolutions of 20 meV and 0.2°, respectively. During the experiment, the sample was maintained in the UHV system under a pressure better than 1 × 10^−10^ torr, and the sample temperature was kept at 10 K. A fresh surface of Bi_2_O_2_Se single crystal for the ARPES measurement was obtained by cleaving the sample in situ along its natural (001) cleavage plane.

### Scanning tunneling microscopy/spectroscopy

The STM/STS experiments were carried out in UHV environment. Bi_2_O_2_Se single crystals were glued onto highly doped silicon wafers and cleaved in situ at room temperature. Cleaved samples were transferred to a cryogenic stage kept at 77 K for STM/STS experiments. Chemically etched tungsten (W) tips were used for both imaging and tunneling spectroscopy. The tungsten tip was calibrated with the surface states of silver islands on Si(111)–7 × 7. Lock-in technique was used to obtain *dI*/*dV* curves. A 5-mV modulation signal at 991 Hz was applied to the sample together with the DC sample bias.

### Ab initio calculations

To resolve the bulk band structure of Bi_2_O_2_Se, we performed the first-principles calculations about Bi_2_O_2_Se. The density functional theory calculations were performed by using the Vienna Ab initio Simulation Package (VASP), with core electrons represented by projector-augmented wave potential (*28*). The plane-wave basis set with an energy cutoff at 400 eV was applied. To obtain accurate band structures, the modified Becke-Johnson exchange potential (*29*) was adopted for the exchange-correlation functional. A *k*-point grid of 35 × 35 × 13 was used for the BZ sampling. Moreover, to identify the exact electronic properties of the surface pattern, we performed first-principles calculations on the Bi_2_O_2_Se slab model.

## Supplementary Material

http://advances.sciencemag.org/cgi/content/full/4/9/eaat8355/DC1

## SUPPLEMENTARY MATERIALS

Supplementary material for this article is available at http://advances.sciencemag.org/cgi/content/full/4/9/eaat8355/DC1 Section S1. SdH quantum oscillation and effective mass in Bi_2_O_2_Se bulk crystal Section S2. Statistical result of Se-atom coverage on the cleaved Bi_2_O_2_Se surface Section S3. Determination of the high-symmetry points along *k*_*z*_ and bulk band structure of Bi_2_O_2_Se Section S4. Potassium doping and the structure of electron pocket Section S5. Fitting of the electron and hole pockets Section S6. Calculation on the formation of surface dimer Section S7. Monte Carlo simulation and analysis of STM image Section S8. Density functional theory calculation on half Se coverage surface Fig. S1. SdH quantum oscillation and effective mass. Fig. S2. STM spectra with atomic resolution in different regions. Fig. S3. Photon energy–dependent ARPES measurements. Fig. S4. Potassium doping and the structure of electron pocket. Fig. S5. Fitting of electron and hole pockets. Fig. S6. Slab model used for the calculation on the formation energies of different Se-atom and vacancy configurations. Fig. S7. Monte Carlo simulation and analysis of STM image. Fig. S8. Theoretical calculation of half Se coverage surface.
