# Supplementary material for: Electronic structures and unusually robust bandgap in an ultrahigh-mobility layered oxide semiconductor, Bi2O2Se
Source: Sci Adv. 2018 Sep 14;4(9):eaat8355. doi: 10.1126/sciadv.aat8355 (PMC6140625; doi:10.1126/sciadv.aat8355)
Supplement: http://advances.sciencemag.org/cgi/content/full/4/9/eaat8355/DC1 [file supp_4_9_eaat8355__index.html]

Science Advances | Science Advances

## Supplementary Materials

**This PDF file includes:**

- Section S1. SdH quantum oscillation and effective mass in Bi2O2Se bulk crystal
- Section S2. Statistical result of Se-atom coverage on the cleaved Bi2O2Se surface
- Section S3. Determination of the high-symmetry points along *k**z* and bulk band structure of Bi2O2Se
- Section S4. Potassium doping and the structure of electron pocket
- Section S5. Fitting of the electron and hole pockets
- Section S6. Calculation on the formation of surface dimer
- Section S7. Monte Carlo simulation and analysis of STM image
- Section S8. Density functional theory calculation on half Se coverage surface
- Fig. S1. SdH quantum oscillation and effective mass.
- Fig. S2. STM spectra with atomic resolution in different regions.
- Fig. S3. Photon energy–dependent ARPES measurements.
- Fig. S4. Potassium doping and the structure of electron pocket.
- Fig. S5. Fitting of electron and hole pockets.
- Fig. S6. Slab model used for the calculation on the formation energies of different Se-atom and vacancy configurations.
- Fig. S7. Monte Carlo simulation and analysis of STM image.
- Fig. S8. Theoretical calculation of half Se coverage surface.

Download PDF

**Files in this Data Supplement:**

- Adobe PDF - aat8355\_SM.pdf
