# Supplementary material for: Electronic structures and unusually robust bandgap in an ultrahigh-mobility layered oxide semiconductor, Bi2O2Se
Source: Sci Adv. 2018 Sep 14;4(9):eaat8355. doi: 10.1126/sciadv.aat8355 (PMC6140625; doi:10.1126/sciadv.aat8355)
Supplement: http://advances.sciencemag.org/cgi/content/full/4/9/eaat8355/DC1 [file aat8355_SM.pdf]

Science Advances
AAAS

advances.sciencemag.org/cgi/content/full/4/9/eaat8355/DC1

Supplementary Materials for

Electronic structures and unusually robust bandgap in an ultrahigh-mobility layered oxide semiconductor, Bi_2O_2Se

Cheng Chen, Meixiao Wang, Jinxiong Wu, Huixia Fu, Haifeng Yang, Zhen Tian, Teng Tu, Han Peng, Yan Sun, Xiang Xu, Juan Jiang, Niels B. M. Schröter, Yiwei Li, Ding Pei, Shuai Liu, Sandy A. Ekahana, Hongtao Yuan, Jiamin Xue, Gang Li, Jinfeng Jia, Zhongkai Liu, Binghai Yan, Hailin Peng*, Yulin Chen*

*Corresponding author. Email: yulin.chen@physics.ox.ac.uk (Y.C.); hlpeng@pku.edu.cn (Hailin Peng)

Published 14 September 2018, *Sci. Adv.* **4**, eaat8355 (2018)
DOI: 10.1126/sciadv.aat8355

This PDF file includes:

Section S1. SdH quantum oscillation and effective mass in Bi_2O_2Se bulk crystal
Section S2. Statistical result of Se-atom coverage on the cleaved Bi_2O_2Se surface
Section S3. Determination of the high-symmetry points along k_z and bulk band structure of Bi_2O_2Se
Section S4. Potassium doping and the structure of electron pocket
Section S5. Fitting of the electron and hole pockets
Section S6. Calculation on the formation of surface dimer
Section S7. Monte Carlo simulation and analysis of STM image
Section S8. Density functional theory calculation on half Se coverage surface
Fig. S1. SdH quantum oscillation and effective mass.
Fig. S2. STM spectra with atomic resolution in different regions.
Fig. S3. Photon energy-dependent ARPES measurements.
Fig. S4. Potassium doping and the structure of electron pocket.
Fig. S5. Fitting of electron and hole pockets.
Fig. S6. Slab model used for the calculation on the formation energies of different Se-atom and vacancy configurations.
Fig. S7. Monte Carlo simulation and analysis of STM image.
Fig. S8. Theoretical calculation of half Se coverage surface.

Section S1. SdH quantum oscillation and effective mass in Bi_2O_2Se bulk crystal

As illustrated in fig. S1a, the Bi_2O_2Se bulk crystal exhibits a very high residual-resistance ratio (RRR, $R_{xx,\,300\,K}/R_{xx,\,2\,K}$) of 585, which is about one order higher than that of the typical CVD-grown 2D Bi_2O_2Se crystal (~60), thereby resulting in a superior Hall mobility at low temperature. We note that the metallic R_{xx}-T behavior here results from the residual carriers - which can be removed by electric gating, as can be seen in *Ref. 19*, where the Bi_2O_2Se field effect transistor shows an insulating behavior with top gating at room temperature (Fig. 4(b) of *Ref. 19*). The long mean free path of the charge carriers in Bi_2O_2Se bulk crystal enables the observation of SdH oscillation at relatively low magnetic field. fig. S1b shows the temperature-dependent Shubnikov-de Haas (SdH) oscillation ranged from 2 to 16 K. To estimate the in-plane cyclotron effective mass m^*, the SdH oscillation amplitude $\Delta R_{xx}/R_0$ was plotted as a function of temperature T using the Lifshitz-Kosevich formula as follows

$$\Delta R_{xx} = 4R_0 \exp\left(\frac{-4\pi^3 k_B T_D}{h\omega_c}\right) \frac{4\pi^3 k_B T / h\omega_c}{\sinh(4\pi^3 k_B T / h\omega_c)}$$

where $\omega_c = eB/m^*$. The effective masses were estimated to be both $0.16 \pm 0.02\ m_0$ (m_0 is the mass of a free electron) at an external magnetic field of 6.02 and 6.69 T (fig. S1(c-d)), showing excellent consistency with the ARPES measurements and theoretical calculations.

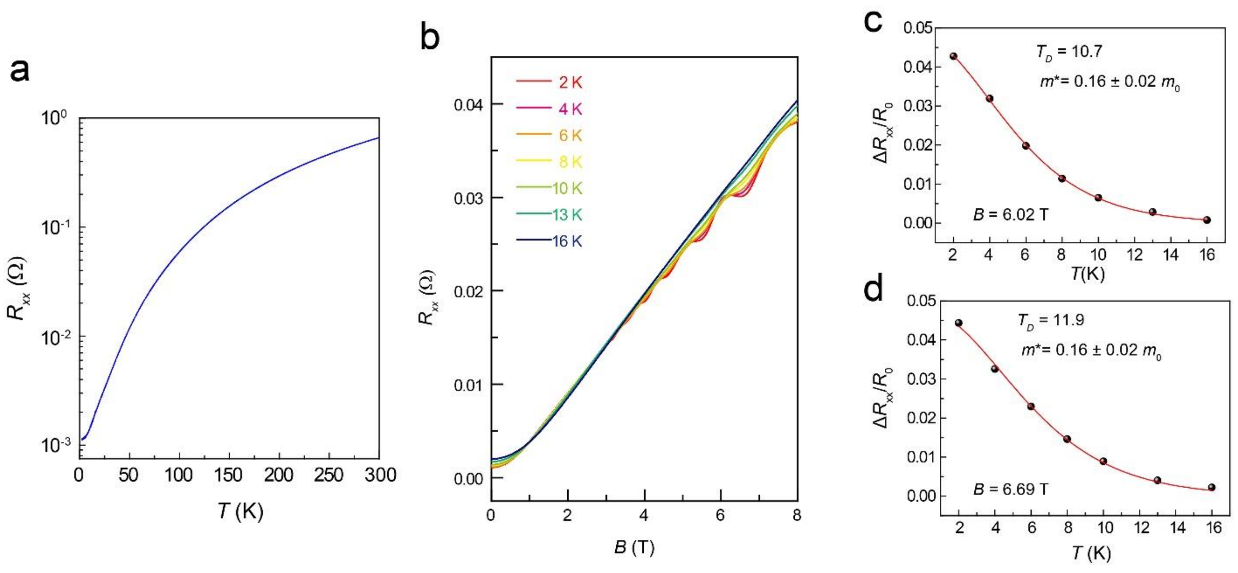

Fig. S1. SdH quantum oscillation and effective mass. a, Resistance as a function of temperature, exhibiting a very high residual-resistance ratio (RRR, $R_{xx,\,300\,K}/R_{xx,\,2K}$) of 585. **b,** Longitudinal resistance (R_{xx}) as a function of the applied perpendicular magnetic field (B) measured at the temperature range from 2 to 16 K. **c-d,** Oscillation amplitude $\Delta R_{xx}/R_0$ as a function of temperature T using the Lifshitz-Kosevich formula fitting at 6.02 T and 6.69 T, respectively.

Section S2. Statistical result of Se-atom coverage on the cleaved Bi_2O_2Se surface

As the Bi-Se interlayer interaction along *c*-axis is much weaker than that of in-plane Bi-O bonds in layered Bi_2O_2Se, the cleavage of Bi_2O_2Se bulk crystals occurs on the Se-plane. As described in the manuscript, only half of the Se atoms remain on the cleavage surface. To verify that, the surface was carefully examined by STM. As indicated in fig. S2, distance between adjacent bright spots is measured to be ~0.39 nm, consistent with the crystal structure of the Se-Se atomic distance, therefore confirms the Se cleavage plane. Line-shape vacancies of atoms are randomly

distributed on the cleaved surface. All four regions illustrated feature an average ~50 % occupancy of Se atoms on the cleavage surface.

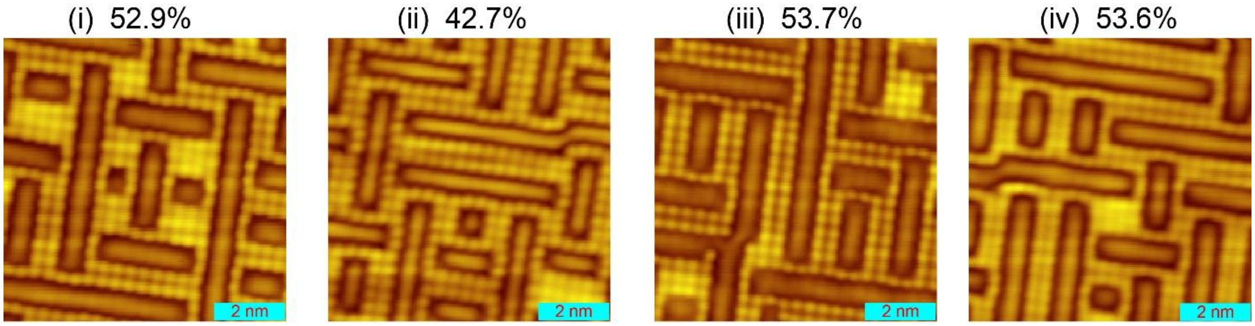

Fig. S2. STM spectra with atomic resolution in different regions. The occupancy rates are calculated, showing an average value around 50%.

Section S3. Determination of the high-symmetry points along k_z and bulk band structure of Bi_2O_2Se

During the photoemission process, the horizontal momentum ($k_{\parallel}$) of band electrons is obtained directly from the free-electrons based on the momentum conservation law. The vertical momentum (k_z) is not conserved. With the free-electron final state approximation and a potential parameter V_0 (also known as the inner potential) describing the energy difference of photoelectrons before and after passing the crystal surface, we can derive the k_z as

$$k_z = \frac{\sqrt{2m_e(E_k \cos^2\theta + V_0)}}{\hbar}$$

where θ is the emission angle and E_k is the kinetic energy of the emitted electron, which satisfies

$$E_k = \text{h}\upsilon - w - E_B$$

where hv is the photon energy, w is the work function of the sample and E_B is the electron binding energy.

As V_0 varies with compounds, we typically perform energy dependent ARPES by using a broad range of photon energies to ensure the full coverage of k_z–span (ideally more than one Brillouin Zone (BZ)), and then use the high-symmetry points in the $k_{||}$-k_z plane of the BZ to identify the exact value of V_0.

As for Bi_2O_2Se, the inner potential was determined to be $V_0 = 12$ eV, according to the k-space conversion of the equal energy contour at Fermi surface (E_F) shown in fig. S3a. According to the appearance of conduction band in the contour (fig. S3a(i)) and the dispersing of wave-like valance bands (fig. S3a(ii)) we can clearly identify the periodicity and determine the high symmetry points. Electronic structure at different k_z values can be accessed with different photon energies. In addition, our ARPES measurements also yielded the detailed evolution of the electronic structures with binding energy, as can be seen in fig. S3b, which will be important for the understanding of the physical properties of Bi_2O_2Se (and its devices) at different doping levels.

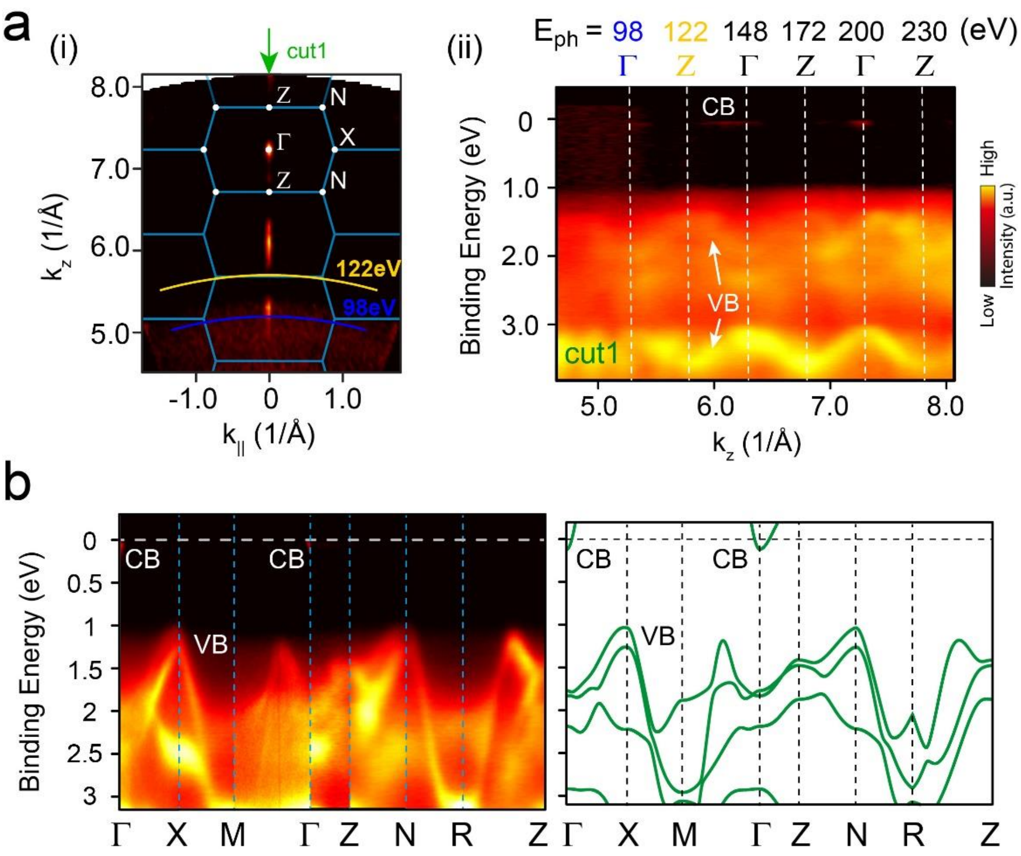

Fig. S3. Photon energy-dependent ARPES measurements. a, (i) Constant energy contour at E_F in the $k_{||}$ and k_z planes of Bi_2O_2Se, in which high symmetry points are labeled. (ii) Band dispersion along Γ–Z direction. High symmetry points and related photon energies are marked above. **b,** General band evolution among high symmetry points, showing excellent agreement with theoretical calculation.

Section S4. Potassium doping and the structure of electron pocket

The electron and hole pocket near the CBM and VBM are essential to the transport properties for n- and p-type Bi_2O_2Se, respectively. As the Fermi-level of the as-grown sample is very close to the CBM which makes its band dispersion difficult to resolve, we used the *in situ* potassium doser (see fig. S4a) inside the vacuum system to introduce the K atoms (thus free electrons) onto the surface of the cleaved sample. We effectively shifted the Fermi-level up by ~160 meV (fig. S4b), which enables us to acquire the detailed dispersion of the parabolic electron pocket, as illustrated in fig. S4c(ii).

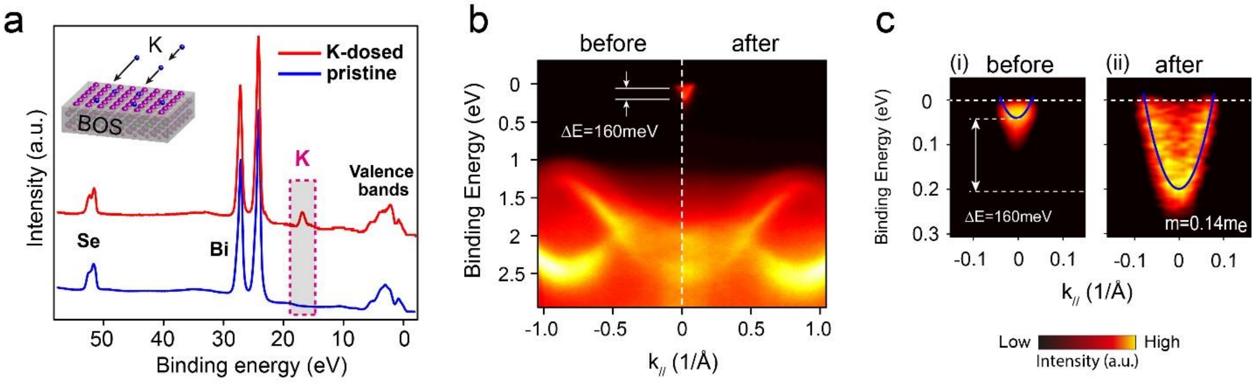

Fig. S4. Potassium doping and the structure of electron pocket. a, Core-level intensity spectra before and after potassium dosing. Inset: Illustration of the dosing process. **b,** Photoemission intensity plots of dispersions along Γ – X direction before and after dosing. **c,** Spectra of conduction band before and after dosing.

Section S5. Fitting of the electron and hole pockets

By detailed analysis, we can fit the dispersion of the pockets to deduce important band parameters (e.g. effective mass and Fermi velocity). As illustrated in fig. S5a, the dispersion of

electron pocket was extracted by finding the peak positions from energy distribution curve (EDC), which was then fitted by a parabolic model $\boldsymbol{E} = \frac{\hbar^2(\boldsymbol{k}-\boldsymbol{k_0})^2}{2\boldsymbol{m}^*} + \boldsymbol{E_0}$ (k_0 and E_0 are the relative momentum and binding energy with reference to the bottom of the conduction or the top of the valence band). The in-plane effective mass of electron $\boldsymbol{m}^*$ is estimated to be $(\mathbf{0.14}\pm\mathbf{0.02})\ \boldsymbol{m_e}$ (m_e is the mass of a free electron), with a Fermi velocity as

$$\boldsymbol{v_F} = \sqrt{\frac{2|\boldsymbol{E}-\boldsymbol{E_0}|}{\boldsymbol{m}^*}} = (\mathbf{3.78} \pm \mathbf{0.01}) \times \mathbf{10^6}\sqrt{|\boldsymbol{E} - \boldsymbol{E_0}|(\mathbf{eV})}\,\mathbf{m}/\boldsymbol{s}$$

Similarly, as illustrated in fig. S5b, the effective mass of hole pockets can also be extracted. Note that there are two close-by hole pockets around X point, so the EDCs are fitted by two Lorentz peaks. Hence, the in-plane effective mass is estimated to be $(-\mathbf{2.41}\pm\mathbf{0.02})\ \boldsymbol{m_e}$ along Γ – X direction, as well as $(-\mathbf{0.30}\pm\mathbf{0.02})\ \boldsymbol{m_e}$ along X – M direction.

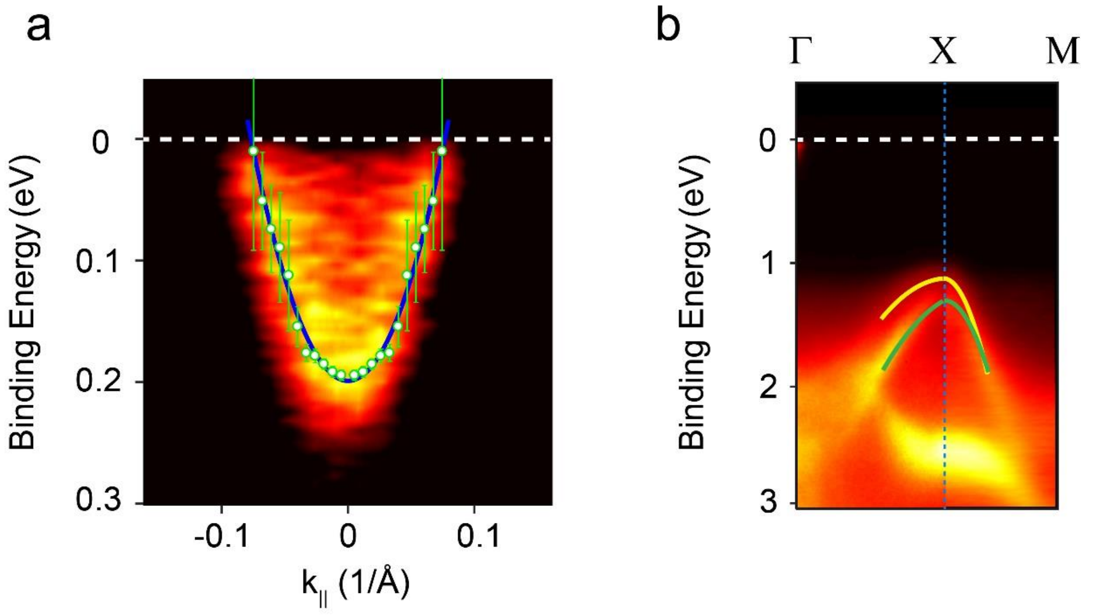

Fig. S5. Fitting of electron and hole pockets. a, Photoemission spectrum of electron pocket located around the Γ point as well as the fitting result. Green dots mark the peak positions of each EDC, while the blue solid line indicates the parabolic fitting result of these peak positions. **b,** Photoemission spectrum of hole pockets around the X point and related fitting result, using the same fitting process.

Section S6. Calculation on the formation of surface dimer

On the cleavage surface of Bi_2O_2Se crystal, both Se atoms and vacancies dimerize and form 2×n structures (Fig. 4a in manuscript). To understand these results, we performed *ab-initio* calculation to estimate the formation energies of different Se-atom and vacancy configurations, by using a slab model, as illustrated in fig. S6. Considering the 4-fold symmetry of the crystal, only x direction is considered here (configuration extended along y). The formation energy was calculated as following

$$E_c = (E_{Se-atom\ chain+substrate} - E_{substrate})/N_{Se}$$

The configuration illustrated in the figure (Se-Se dimer with vacancy on each side) was calculated to have the lowest formation energy, therefore is most favorable. This explains the formation of Se-atom and vacancy dimers on the cleavage surface of Bi_2O_2Se bulk crystals.

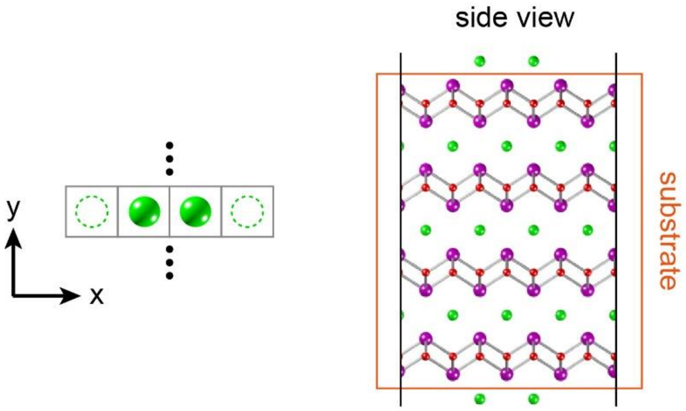

Fig. S6. Slab model used for the calculation on the formation energies of different Se-atom and vacancy configurations.

Section S7. Monte Carlo simulation and analysis of STM image

As discussed in the manuscript, the chain of Se-Se dimer with one vacancy on each side proves to be the most energy favorable configuration. Therefore, 4×4 square building blocks (extended configuration in 2D space) was used for a Monte Carlo simulation (Tile model) to simulate the patterns on the half-Se-coverage surface (fig. S7a). The assumption is that the building blocks have equal possibility to distribute along *x* or *y* direction, considering the 4-fold symmetry of the crystal. A 50×50 matrix is firstly generated with each element containing a random value (either 0 or 1 with equal possibility). Then the elements containing 0 or 1 were replaced by building blocks along *x* or *y* direction respectively. The simulated pattern is illustrated in fig. S7a, which highly matches the real situation.

To have a quantitative comparison, the possibility of different lengths of vacancy dimers was deduced from both simulated and real images. For the real STM image, as illustrated in fig. S7b, original image was firstly binarized with the local thresholding method. Different vacancy dimer chains were separated by computing the area connectivity. Length of each vacancy dimers chain was then calculated and a statistic distribution was obtained (Fig. 4b(ii) in manuscript).

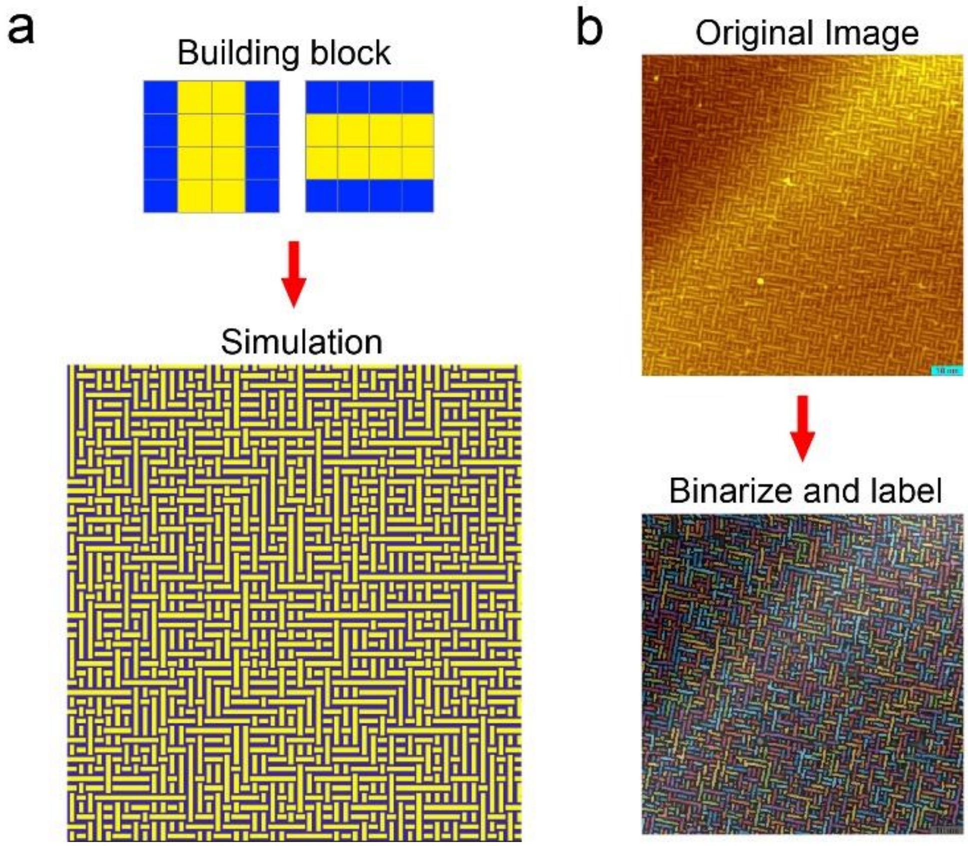

Fig. S7. Monte Carlo simulation and analysis of STM image. a, Monte Carlo simulation of surface patterns. **b,** Analysis method for STM image to obtain statistical result.

Section S8. Density functional theory calculation on half Se coverage surface

In order to identify the exact electronic properties of the surface pattern, we performed first-principles calculations on the Bi_2O_2Se slab model. The Bi_2O_2Se slab (fig. S8) contains four $[Bi_2O_2]^{2+}$ layers and five Se^{2-} layers and corresponds to a 4 × 1 surface supercell. The top and bottom Se layers are symmetrical with alternate Se-Se dimers and Se-Se vacancies along x-axis, consistent with the surface pattern observed in experiment. A vacuum region of >15 Å thick along the surface normal direction is sufficiently large to eliminate artificial interactions between periodic images. Geometry optimization was carried out until the residual force on each atom was less than 0.01 eV/Å. To obtain the accurate density of states, a k-point grid of 8 × 32 × 1 was used for the Brillouin zone sampling. The calculated band structure is illustrated in fig. S8b, where the contributions from Bi-top and Se-top atoms are labeled. The Density Of States(DOS) were projected to the top three Bi atoms for the Bi-top region, and to the top Se atom and underlying two Bi atoms for the Se-top region, as shown in the manuscript (Fig. 4c(ii)). In addition, we note that the seemingly direct energy gap at the Γ point in fig. S8 is due to the band folding of the supercell structure.

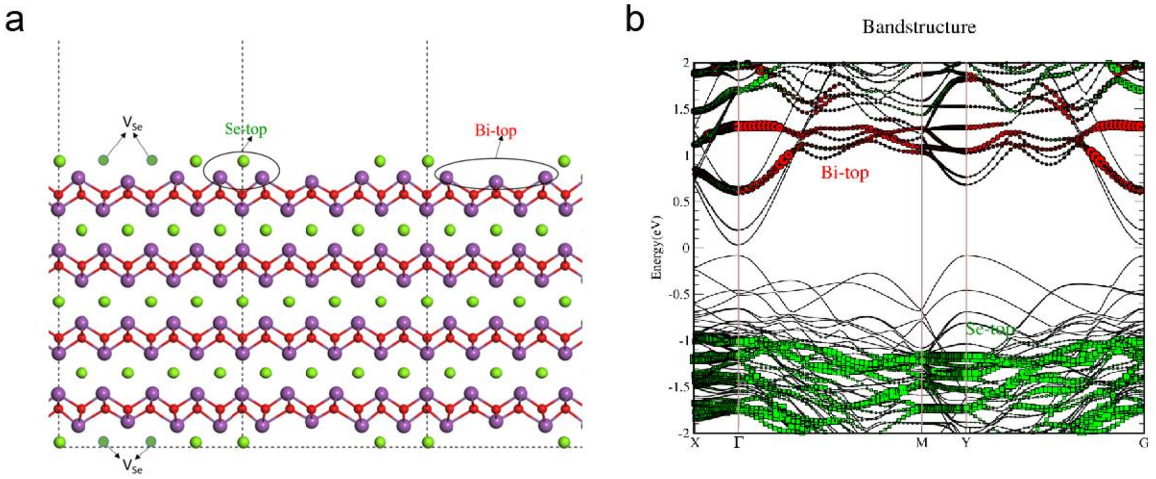

Fig. S8. Theoretical calculation of half Se coverage surface. a, Slab model of the half-Se-coverage surface condition of cleaved Bi_2O_2Se. **b,** Calculated band structure, contributions of Bi-top and Se-top atoms are labeled with red and green colors respectively.
